# Destitute and dying: interventions and models of palliative and end of life care for homeless adults – a systematic review

**DOI:** 10.1136/spcare-2024-004883

**Published:** 2024-08-17

**Authors:** Megan Rose Coverdale, Fliss Murtagh

**Affiliations:** 1 Wolfson Palliative Care Research Centre, Hull York Medical School, University of Hull, Kingston upon Hull, UK

**Keywords:** Palliative Care, Terminal care, Supportive care, Hospice care, Quality of life

## Abstract

**ABSTRACT:**

**Background:**

Homeless adults experience a significant symptom burden when living with a life-limiting illness and nearing the end of life. This increases the inequalities that homeless adults face while coping with a loss of rootedness in the world. There is a lack of palliative and end of life care provision specifically adapted to meet their needs, exacerbating their illness and worsening the quality of their remaining life.

**Aim:**

To identify interventions and models of care used to address the palliative and end of life care needs of homeless adults, and to determine their effectiveness.

**Methods:**

Standard systematic reviewing methods were followed, searching from 1 January 2000 the databases: Ovid MEDLINE, EMBASE, SCOPUS, Web of Science, CINAHL and PsycInfo. Results were reported following Preferred Reporting Items for Systematic Reviews and Meta-Analyses guidelines and described using a narrative synthesis. Study quality was assessed using Hawker’s Quality Assessment Tool.

**Results:**

Nine studies primarily focused on: education and palliative training for support staff; advance care planning; a social model for hospice care; and the creation of new roles to provide extra support to homeless adults through health navigators, homeless champions or palliative outreach teams. The voices of those experiencing homelessness were rarely included.

**Conclusion:**

We identified key components of care to optimise the support for homeless adults needing palliative and end of life care: advocacy; multidisciplinary working; professional education; and care in the community. Future research must include the perspectives of those who are homeless.

**WHAT IS ALREADY KNOWN ON THIS TOPIC**

- Use of palliative care services by the homeless population is limited; there is a considerable lack of end of life care provision specifically adapted to meet their biopsychosocial needs.

**WHAT THIS STUDY ADDS**

- This systematic review provides a detailed understanding of the nature of interventions and models of care currently used to deliver palliative and end of life care to homeless adults, and what interventions are most effective to enhance the support for these marginalised individuals.

**HOW THIS STUDY MIGHT AFFECT RESEARCH, PRACTICE OR POLICY**

- Key components have been identified which are most important for optimising the delivery of palliative and end of life care to homeless adults: advocacy; multidisciplinary working; professional education; and care integrated into the community settings where the homeless population is based. Future research must include the perspectives of those who are homeless, build on the components which we know work, and address the sustainability of these interventions and models of care.

## Background

 The numbers and needs of people experiencing homelessness while living with a life-limiting illness are increasing, yet these marginalised individuals are restricted from mainstream health and social care, despite often having the greatest needs; this is a pertinent issue that must be addressed within palliative and end of life care.^1^ The number of people experiencing homelessness in the UK is rapidly rising; Shelter^2^ reports that 1/182 people are homeless, with over 3000 rough sleeping every night. Similarly, rates of homelessness within European countries including Germany, Spain and Ireland are also rising: recent data from FEANTSA^3^ (the European Federation of National Organisations Working with the Homeless) report rising levels of homelessness within these countries, reaching 262 645, 28 552 and 11 632, respectively. However, these figures are likely an underestimation due to ‘hidden homelessness’ in which an individual is homeless but missing from the data.

Homelessness, according to Somerville,^4^

is not just a matter of lack of shelter or lack of abode, a lack of a roof over one’s head. It involves deprivation across a number of different dimensions—physiological (lack of bodily comfort or warmth), emotional (lack of love or joy), territorial (lack of privacy), ontological (lack of rootedness in the world, and anomie [a theory in which purpose and goals cannot be achieved due to lack of means^5^]) and spiritual (lack of hope, lack of purpose).

Elements of the ETHOS light criteria^6^ have been adopted within this review to describe the different types of homelessness (table 1). Many homeless adults suffer with trimorbidity, explained by Vickery *et al*^7^ as ‘a subset of multimorbidity representing overlap of physical health, mental health, and substance use conditions’; this can make caring for these individuals complex and challenging. Health inequalities are evident for this ostracised community, and life expectancy is exceedingly low: 43 years for women and 45 years for men, compared with the UK national average of 83 years and 79 years, respectively.^8^ Most notably, deaths within the homeless community are continuing to rise annually.^9^

**Table 1:** The spectrum of homelessness

Operational category	Living situation	Definition
People living rough	Public spaces/external spaces	Living in the streets or public spaces without a shelter that can be defined as living quarters
People in emergency accommodation	Overnight shelters	People with no place of usual residence who move frequently between various types of accommodation
People living in accommodation for the homeless	Homeless hostels and temporary accommodation	Where the period of stay is time-limited, and no long-term housing is provided
People living in non-conventional dwellings due to lack of housing	Mobile homes, non-conventional buildings	Where the accommodation is used due to a lack of housing and is not the person’s usual place of residence
Homeless people living temporarily in conventional housing with family and friends (due to lack of housing)	Conventional housing, but not the person’s usual place of residence	Where the accommodation is used due to a lack of housing and is not the person’s usual place of residence

Adapted from ETHOS light criteria.^6^

Somerville highlights the crucial need for a holistic approach to care for homeless adults, yet numerous hurdles exist in the delivery of good palliative and end of life care for this population. Homeless adults often experience a large symptom burden near the end of life, particularly pain, worry, sadness and exhaustion.^10^ Many also have growing mistrust in healthcare professionals and underuse healthcare services due to fear of stigmatisation, discrimination and perceived healthcare prejudice. There is a considerable lack of palliative and end of life care provision specifically adapted to meet the biopsychosocial needs of the homeless population; this exacerbates the illness burden for these destitute individuals and worsens the quality of their remaining lifetime.

A systematic review was undertaken to identify the strengths and gaps in the delivery of palliative and end of life care to homeless adults, and make recommendations to bridge these gaps, with the aim of improving health and social care practice. Our review question aimed to determine what interventions and models of care are used to address the palliative and end of life care needs of adults who are experiencing homelessness, and are they effective? The objectives for adults experiencing homelessness, and needing palliative and end of life care, were to: (1) describe the interventions and models of care and (2) consider the strengths and gaps of the interventions and models of care, and discuss their effectiveness.

## Methods

Preliminary searching was first undertaken using the Database of Systematic Reviews, the Database of Abstracts of Reviews of Effects and PROSPERO. We identified no similar systematic reviews, however, a relevant scoping review by James *et al*^11^ reports that the provision of palliative care to homeless adults is complex with many barriers hindering the delivery of quality care.

We conducted a systematic review using a standard methodological framework, adapted from the Centre for Reviews and Dissemination^12^ (CRD) on how to undertake systematic reviews in healthcare. We followed the PEOS (Population, Exposure, Outcome and Study Design) framework to build a focused research question (table 2). Case series and case reports, commentary, review and opinion pieces were excluded due to the high potential for bias within these types of study designs. Research which did not report on interventions or models of care, or include homeless adults for at least 50% of their study population was also excluded. Results were reported following Preferred Reporting Items for Systematic Reviews and Meta-Analyses reporting guidelines.^13^

**Table 2:** PEOS framework

Population	Adults, aged 18 and over, who are homeless and living with a terminal illness, or the professionals caring for them
Exposure	Interventions and models of care used
Outcome	To determine the components and effectiveness of the interventions and models of care in optimising care for homeless adults, measured as improvement in any of the domains: quality of life, well-being, symptom improvement, efficiency and knowledge
Study Design	Qualitative and quantitative study designs

Our search strategy (table 3) was independently reviewed and refined by a librarian with expertise in information skills. Systematic searches were conducted on electronic databases using MeSH terms employing Boolean logical operators of “AND” and “OR”, in addition to free text searches (identified as key words). Truncations of words using an * was undertaken to enable the inclusion of multiple endings of the specific term. We limited the search criteria to “English language”. Online databases were searched on 22 November 2023 for articles published from 1 January 2000 using Ovid MEDLINE, EMBASE, SCOPUS, Web of Science, CINAHL and PsycInfo.

**Table 3:** Search strategy

Database: Ovid MEDLINEDate 22 November 2023	Search terms	Number of records
1	palliative OR “end-of-life” OR “end of life” OR “advance* care plan*” OR “attitude to death” OR terminal* OR hospice* OR “life support care” OR death OR dying OR bereav*	1 631 931
2	homeless* OR “ill-housed” OR “ill housed” OR “shelterless” OR “street people” OR “street person” OR unhoused OR roofless OR destitute	17 075
3	1 AND 2	831
4	Limit 3 to English Language and published from 01/01/2000	694

All identified citations were uploaded into the bibliographic software, EndNote21, and duplicate studies were removed. During initial screening, one author (MRC) oversaw the screening of study titles and abstracts; discussion with the second author (FM) was undertaken on 5% of studies during this stage to determine their relevance to the research question. One reviewer undertook full-text screening of studies (MRC); 30% of studies at this stage were discussed with the second author (FM) to determine suitability for inclusion. Data were extracted into tabular format on the design, context, quality and effectiveness of interventions and models of care.

Quality assessment of individual studies was formally undertaken using Hawker’s Quality Assessment Tool for Qualitative Studies.^14^ Nine domains within each individual study were assessed and categorised as being of good, fair, poor or very poor quality. The minimum score that could be achieved for each paper using this tool was 9, the maximum 36. High, medium and low quality studies were determined based on their cumulative score across the nine domains, ranging between 30–36, 24–29 and 9–23, respectively.

A meta-analysis was not completed due to the heterogeneity of studies; instead, we undertook a narrative synthesis due to its appropriateness for organising and summarising the main findings from a varied body of research. We used formal guidance by Popay *et al*^15^ for conducting a narrative synthesis and the following steps were addressed: ‘developing a theory of how the intervention works, why and for whom; developing a preliminary synthesis of findings of included studies; exploring relationships in the data; assessing the robustness of the synthesis’. We selected thematic analysis as the best method to describe the interventions and models of care, and consider their strengths, gaps and effectiveness.

## Results

### Study selection

**Figure 1:**
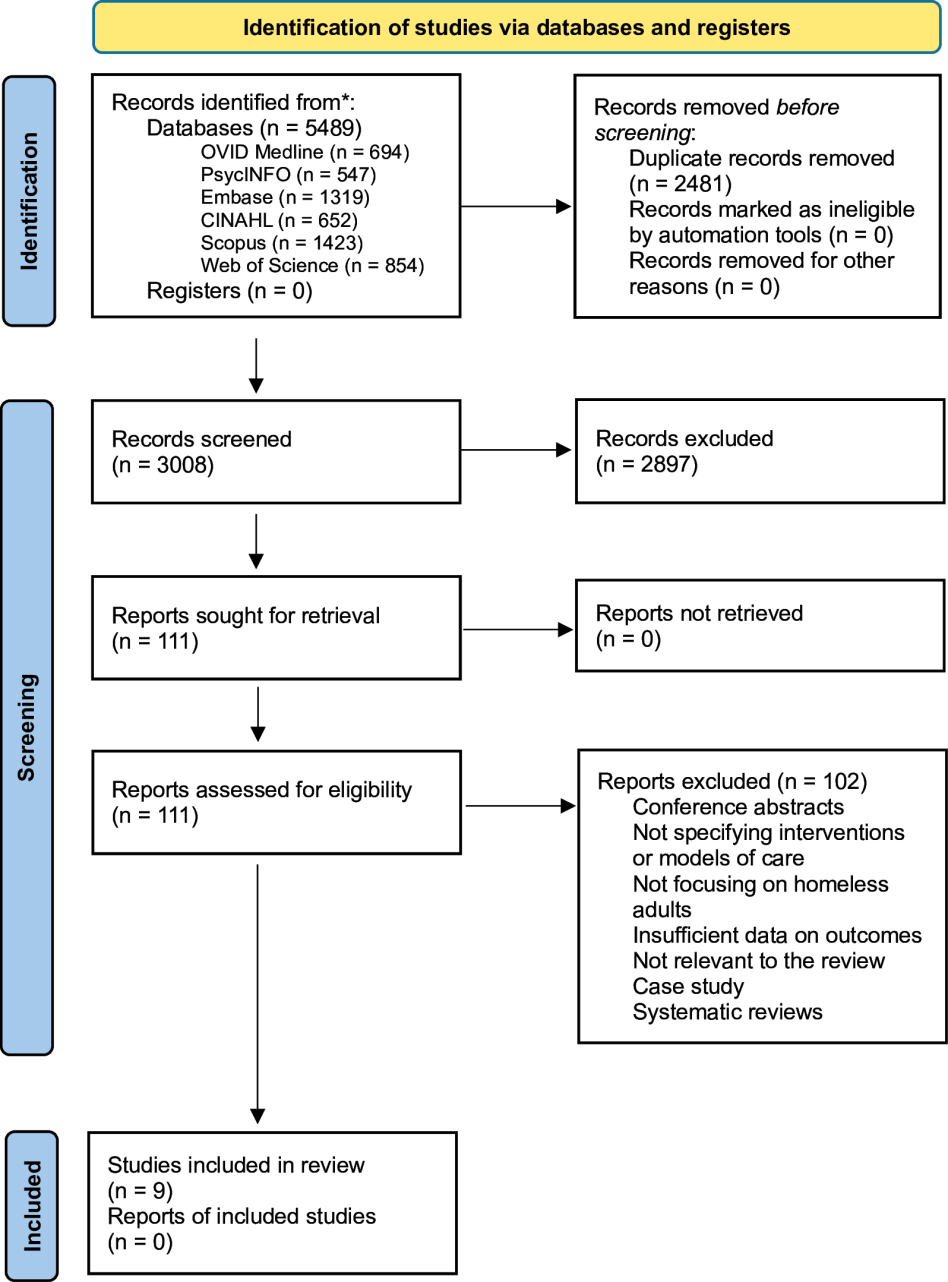
Preferred Reporting Items for Systematic Reviews and Meta-Analyses flow diagram.^13^

The search was conducted in November 2023. 5487 studies were initially identified. After screening and full-text review, nine studies were included (figure 1).

### Study characteristics

Three included studies were qualitative,^16^,^18^ one was mixed methods,^19^ three were service evaluations/improvements,^20^,^22^ one was a retrospective cohort study^23^ and one was a randomised control trial.^24^ Different interventions and models of care and whether they worked were mainly reported qualitatively, however, four studies included quantitative reporting.^19 20 23 24^ Qualitative data were collected using questionnaires,^19 24^ semi-structured interviews,^18^ monthly reporting via email and telephone,^18^ focus group interviews^19^ and photovoice exploration in which participants photographed components of a model of care found to be most meaningful to them.^17^ Quantitative data were collected from tabulation of key performance indicators achieved,^20^ review of patient medical notes,^23^ evaluation of baseline and outcome questionnaires^19^ and uptake of an intervention.^16 24^ Results from two studies did not specify how they were collected.^21 22^ The locations of these included studies were the UK,^18 19 21^ Canada^20 22 23^ and the USA.^16 17 24^ All interventions and models of care were instigated between the years 2001 and 2021.

Sample sizes were only reported in some studies. Where documented, numbers of participants ranged from 3 to >150. Five study populations primarily reported from professionals^18^,^22^ with roles including non-clinical hostel staff, palliative care doctors, palliative care nurses, social workers and community nurses. Four study populations focused primarily on the care of homeless adults.^16 17 23 24^ Financial reimbursement was provided within two studies to homeless adults for their participation.^17 24^ Only one study focused on the experiences of a model of care directly from the perspective of the homeless adults involved; this was through interpretation of photos taken by patients during their stay in a social hospice.^17^

Demographics of homeless adults were not always reported. However, in studies that did document homeless adult characteristics, most were of white ethnicity; two study populations predominantly consisted of black homeless adults.^16 24^ There was a significant male preponderance in all the homeless populations studied; one study included transgender adults.^23^ Homeless adults involved in this review were at different stages of their disease trajectories: one study focused on the transfer of terminally ill homeless individuals into a hospice to die in their preferred place of death^23^; another focused on the provision of care to adults with a predicted life expectancy of under 6 months,^17^ and a further study emphasised engagement with homeless adults living with a high degree of frailty.^22^

Most homeless individuals were hostel-based. Only two papers included homeless adults that were rough sleeping, couch surfing or vulnerably housed within other accommodations beyond the realms of a shelter.^17 23^ Most homeless adults were aged under 65 years due to age limits of sheltered accommodation restricting acceptance beyond this age.^18^ Many were living with trimorbidity (overlap of poor physical health, poor mental health and substance use conditions); substance use is a common barrier to being accepted into hostels and hospices.^16 18 23^ However, in light of this, three studies were inclusive of patients with addiction: two implemented a harm reduction strategy to minimise the adverse effects of substance misuse^22 23^; one explicitly enrolled homeless adults living with a substance abuse diagnosis.^16^

### Quality of included studies

The quality assessment of all included studies can be found within online supplemental table 1. The highest scoring study in this review scored 33/36,^18^ while the lowest scored 17/36.^21^ The highest quality score was awarded to an explorative qualitative study. It described the nature of the intervention and its success in detail using interviews and surveys, completed preintervention and postintervention, to gain insight into its impact. Its methodology was clearly ascribed with minimal areas for bias identified. The lowest quality study was a service evaluation on a nurse led homeless project. Despite the model working well to deliver its outcomes, the study had poor internal validity with limited description of its methodology and sampling; and little reporting of limitations.

### Nature of the interventions/models of care

The nature of the interventions and models of care identified are explored in table 4, including specification of the types of homelessness included. The included studies primarily focused on education and palliative training for support staff,^18 19 21 22^ advance care planning,^16 19 23 24^ the creation of new roles to provide extra support to homeless individuals via the introduction of health navigators,^20^ homeless champions^18^ or palliative outreach teams.^22^ One study focused on the implementation of a social model of hospice care based within the community.^17^ Three studies were undertaken within hostels,^16 18 19^ two within hospices^17 23^ and four were embedded directly into the community as in-reach models of care.^20^,^2224^

**Table 4:** The nature of the interventions and models of care

Intervention/model of car	Setting, duration, location, mode of delivery, delivered by?	Intervention recipients	Components of the intervention/model of care
Advance directive completion^24^	Setting: hostel.Duration: 9 months.Location: USA.Mode of delivery: face to face.Delivered by social workers	Homeless adults.Type of homelessness: residents attending emergency shelters, 24-hour shelters, day programmes, treatment programmes and case management programmes.Age range: 18–74 years old.Gender: 74% male.Ethnicity: white, black, native American, Asian, mixed race.Education: 31% did not complete high school.Prognosis: not reported.Main condition: not reported.Substance use: not reported	Counsellor directed completion of an advance directed versus self-directed completion of an advance directive. The advance directive form was specifically designed to address the end-of-life needs of homeless adults. Participants were provided with documentation detailing what advance care planning is following enrolment
Healthcare navigator^20^	Setting: community.Duration: 1 year.Location: Canada.Mode of delivery: face to face.Delivered by one healthcare navigator with a background in social work	Homeless adult demographics were not reported	Duties included signposting and assisting homeless adults with multiple activities of daily living, including attending medical appointments, referring to hostels, accessing food banks. Advocating for these marginalised individuals when appropriate including instigating advance care planning
Social hospice model^17^	Setting: hospice.Duration: 8 months.Location: USA.Mode of delivery: face to face.Delivered by hospice staff and local volunteers	Homeless adults.Type of homelessness: hospice based.Age range: 31–54 years old.Gender: two males, one female.Ethnicity: whiteEducation: not reported.Prognosis: <6 months life expectancy.Main condition: two people had liver failure; one person had cancer.Substance use: not reported	Residents received personalised 24-hour care and support. Living within a social environment united residents and created a shared sense of belonging. This enhanced social interaction and emotional well-being
Medical student led intervention on the completion of advance directives^16^	Setting: hostel.Duration: not reported.Location: USA.Mode of delivery: face to face.Delivered by one medical student	Homeless adults.Type of homelessness: shelter based.Age: 23–55 years old.Gender: all males.Ethnicity: eight African American, one Caucasian, one Latino.Education: not reported.Prognosis: not reported.Main condition: not reported.Substance use: all recipients had a substance abuse diagnosis	Free assistance in the completion of an advance directive, with prior in person counselling on what an advance directive entails. The provision of written information for reference was also given to participants
The Palliative Education and Care for the Homeless (PEACH) programme^22^	Setting: community.Duration: 2014 to present.Location: Canada.Mode of delivery: face to face.Delivered by a collaboration of multiple health and social care disciplines, including physician, nurse, psychiatrist, community support workers	Homeless adult demographics were not reported	Four key domains were addressed when engaging with homeless adults (harm reduction, trauma informed care, intersectionality and antioppression, interprofessional approach to care). Involvement within education and research on reducing inequalities of this marginalised population was also a key component
Shelter based hospice for the homeless^23^	Setting: hospice.Duration: 26 months.Location: Canada.Mode of delivery: face to face.Delivered by registered nurses, community and hospital doctors, hostel staff	Homeless adults.Type of homelessness: shelter based.Age: 49 years old (mean)Gender: 25 males, 2 females, 1 transgender.Ethnicity: not reported.Education: not reported.Prognosis: terminally ill.Main condition: liver disease 43%, HIV/AIDS 25%, malignancy 25% and other 8%.Substance use: 82% had an addiction to drugs or alcohol	Patients resided within a designated 15 bed area and received comprehensive 24/7 healthcare and palliative care consultation to optimise symptoms. Religious needs were addressed, end of life issues were discussed and continuity of care was maintained during the terminal phase of life. Patients were reunited with family when and where possible
Education programme^19^	Setting: hostel.Duration: 3 months.Location: UK.Mode of delivery: face to face and online.Delivered by hostel staff (training course material provided by Marie Curie and St. Mungo’s hostels)	Recipients of the intervention were hostel staff with a variety of experience working with homeless adults, ranging from 1 to 16 years	2-day educational course implemented to train and support hostel staff in their duties when working with homeless adults requiring palliative care
The homeless project^21^	Setting: community.Duration: not reported.Location: UK.Mode of delivery: face to face, and online.Delivered by nurses	Recipients of the intervention were front line staff working within supported, temporary and emergency homeless accommodation	Programme which trained staff how to provide outreach support to homeless individuals needing palliative and end of life care. This was achieved through improved awareness of the early identification of patients with deteriorating health, signposting to relevant agencies for additional support, and initiating advance care planning discussions
In-reach support model^18^	Setting: hostel.Duration: 18 months.Location: UK.Mode of delivery: face to face. Delivered by nurses and social care workers (mainly female, with a range of 1–8 years experience working within palliative care)	Homeless adult demographics were not reported	Homeless champions were appointed to work within four hostels and contracted to attend the hostels for two half days per month. Homeless champions were tasked with multiple responsibilities including supporting hostel staff and residents through providing bereavement support, implementing multidisciplinary working into routine practice, signposting to external agencies and directly meeting with residents of concern when needed. The new role of a homeless champion was directly incorporated into the job specification in replace of previous duties

### Effectiveness of the interventions/models of care

Outcomes and effectiveness of the interventions and models of care are reported in table 5 (see online supplemental table 2 for a detailed review of the evidence of effectiveness). Multiagency communication and collaboration were common findings which enhanced the quality of palliative and end of life care provided to homeless adults and reduced care fragmentation among the various professionals involved.^18^,^20^ Advocacy was another common attribute of many interventions, enhancing person centred care for vulnerable adults experiencing homelessness. The introduction of a healthcare navigator, with expertise in social work, enabled the social determinants of health to be targeted for homeless adults in receipt of care.^20^ Similarly, social worker participation in advance directive completion favourably enhanced uptake of the intervention.^24^ Embedding specialist palliative care teams into hostels^18^ helped hostel staff to develop an increased awareness of both the social and healthcare needs of their hostel residents. Through collaboration with social workers and palliative care nurses, hostel staff felt this intervention was invaluable and allowed for the provision of individually tailored, holistic care.^18^

**Table 5:** Outcomes and effectiveness of identified interventions and models of care

Reference, duration, location, study type	Outcomes	Effectiveness
‘Effect of an end-of-life planning intervention on the completion of advance directives in homeless persons’Song *et al*^24^Conducted: November 2007 to August 2008Location: USAStudy type: randomised control trial	Primary outcome: completion of an advance directive within 3 months of enrolment into the study. Advance directives were assessed for their legibility and legality status by multiple independent investigators who were blinded to intervention groups. 70/262 completed the advance directive. 2 were not counted due to illegibility. Higher completion rate in the counsellor guided intervention group: 37.9% compared with the self-directed group:12.8%. P value <0.001.	++
‘The benefits and challenges of embedding specialist palliative care teams within homeless hostels to enhance support and learning: perspectives from palliative care teams and hostel staff’Armstrong *et al*^18^Conducted: December 2018 to June 2020Location: UKStudy type: exploratory qualitative study	Hostel residents reported positively feeling cared for by staff. Staff felt empowered following introduction of homeless champions; there was a shift in mindset on the development of holistic palliative ethos within their practice. Homeless champions improved interagency and multiagency communication and collaboration. Mental health was addressed through the introduction of a death cafe and vigil. Residents were less inclined to blame staff for deaths of their peers; staff and residents supported each other through the grieving process	+++
‘Assessing the impact of a health navigator on improving access to care and addressing the social needs of palliative care patients experiencing homelessness: a service evaluation’Robinson *et al*^20^Conducted: July 2020 to July 2021Location: CanadaStudy type: service evaluation	One social worker assisted a maximum of 50 homeless adults within their service at any one time. 2007 activities completed by the healthcare navigator for adults experiencing homelessness and needing palliative care support. A focused approach to the social determinants of health was undertaken to facilitate equitable care at the end of life	++
‘Supporting homeless people with collaborative palliative and end-of-life care’Speight and Lyons^21^Conducted: 2021Location: UKStudy type: service improvement	Front line staff were engaged to deliver advance care planning with homeless adults; they were understanding of the importance of advance care planning and increased its uptake and completion. Increased number of homeless individuals dying with dignity in preferred place of care with the right support. Three patients died in preferred place of care; four patients were supported in accommodation. Improved front line staff knowledge on the identification of deteriorating patients, palliative and end of life care	+
‘Shelter-based palliative care for the homeless terminally ill’Podymow *et al*^23^Conducted: June 2001 to August 2003Location: CanadaStudy type: retrospective cohort study	Hostel staff were responsible for supervised provision of medication which improved compliance in the homeless population. No increase in substance abuse. Continuity of care in the terminal phase was achieved. Homeless adults were reunited with family when wanted, and where possible. 57% had palliative care consult. 82% died in hospice; 18% of homeless adults transferred to the emergency department for further symptom control at their request. End of life issues were discussed, and religious needs met	+
‘Effect of a medical student-led end-of-life planning intervention in completion of advanced directives among homeless persons’Coulter^16^Conducted: 2016Location: USAStudy type: qualitative study	9/10 homeless adults who attended focus groups signed up to complete an advance directive. 88.8% completion rate	++
‘Evaluation of training on palliative care for staff working within a homeless hostel’Shulman *et al*^19^Conducted: 2018Location: UKStudy type: mixed methods study with pretraining and post-training data collection	All sections of the course were interesting and useful according to hostel staff. Knowledge of palliative care and services available was most improved among hostel staff. Staff gained confidence in supporting their clients; also gained knowledge on how to access support, and how to give support to residents. Hostel staff more open to confront ill health and talk about it with the clients. Enhanced self-awareness of staff to support their own health and well-being. Work related stress was also slightly improved. Improved attitudes and openness of hostel staff to supporting dying patients and accepting of these dying patients remaining in the hostel. However, some staff did not want the hostel to turn into a hospice environment	+
‘Experiences of homeless recipients of social model hospice care: a photovoice exploration’Jensen *et al*^17^Conducted: January to August 2020Location: USAStudy type: qualitative case report	Patient reported outcomes assessed including: physical location to receive care; community involvement in care; spiritual needs addressed via access to a chaplain. Allowed to bring pets on site. Family re-connected with residents. Residents felt ‘at home’ and cared for by staff and other residents. Harm reduction strategy allowed residents to die with dignity in a comfortable, peaceful location. Healthcare needs and medication was provided by certified nurse’s assistants. Certified nurse assistants (CNAs) were onsite 24/7 and befriended residents; this created a sense of community and connection which made residents feel more confident in their caregivers	++
‘Palliative Education and Care for the Homeless (PEACH): a model of outreach palliative care for structurally vulnerable populations’Buchanan *et al*^22^Conducted: 2014 to presentLocation: CanadaStudy type: service evaluation	Social service workers and community workers could directly refer to the PEACH model and at the earliest opportunity. Education was a priority—active role in teaching and offering electives to medical students. Reflective practice was encouraged for all professionals involved which helped them to explore ways in reducing barriers faced by clients in their practice and beyond. Advocacy was a key component of the PEACH programme- high quality, early and integrated palliative care for vulnerable patients. Advocating for food, housing, healthcare. Advocating at the population level to call for societal change was undertaken via teaching and engagement with local government to tackle the issues arising around homelessness and poverty. Focused on research on the palliative needs of homeless individuals. Support was provided on funeral arrangements, leisure activities and reconnecting homeless adults with family members. Grief circles encouraged all professionals involved in the programme to come together and support one another in grieving a client’s death	++

–, did not work; +, worked slightly; ++, worked moderately; +++, worked well.

Educational programmes^19 21^ enhanced the confidence and knowledge of hostel staff on the ethos of palliative care and how to use this within their practice, noting that the training was ‘invaluable’, ‘extremely beneficial’^18^ and ‘empowering’.^21^ However, it was mentioned that the increased workload relating to educational programmes risked potential staff burnout. To minimise this, two interventions encouraged hostel staff to optimise their well-being through use of counselling services and psychological support.^20 22^

Working within supportive environments enabled hostel staff to improve productivity and become more proactive, liaising with colleagues, and challenging external agencies, when needed, to act in the best interests of their residents.^18^ Hostel staff used their new skills to commence discussions on death and dying with hostel residents; they felt empowered having broken down the taboo associated with this subject.^21^ Hostel residents emphasised the beneficial impact of these timely conversations, and positively reported that they felt cared for which reduced fear and anxiety.^18 21^ Early recognition of health deterioration by hostel staff allowed for a prompt transfer of homeless adults to hospices or hospitals, according to their wishes. This allowed homeless adults to die with dignity and in the place of their choosing with appropriate support.^21^

To address the impact of grief and bereavement, spiritual support was offered for both homeless individuals and professionals involved in their care.^23^ A chaplain was available to provide religious counsel in one study.^17^ Grief circles,^22^ death cafes, and vigils were also introduced into hostels.^18^ These interventions were used to help ease the loss of fellow residents and improve psychological well-being within supportive and nurturing environments. Pets were encouraged in one hospice to provide invaluable companionship, unconditional love and comfort to their owners who were living with a terminal illness.^17^

A key challenge faced when engaging homeless adults in end of life discussions was their concurrent addictions to drugs and alcohol; this was a barrier that professionals involved in their care often struggled to overcome.^19^ However, interventions were also identified which actively overcame this struggle and acknowledged the vulnerability of homeless adults, including factors that potentiate their low self-esteem, such as racism, addiction and homophobia.^19 22 23^ Harm reduction strategies were advantageous in caring for homeless adults with trimorbidity, and when coupled with the reduction of pain and other symptoms attributable to terminal conditions, homeless adults were able to gradually reduce their intake of illicit drugs.^23^

Similarly, the utilisation of trauma informed care for homeless adults^22^ (a method which increases professional understanding that many homeless adults have lived through an unsurmountable level of trauma), helped to educate professionals involved in their care as to why they may maintain dependency on substances, despite the severity of their faltering health. Hostel staff was encouraged to build rapport with residents and break down existential barriers (including stigma for selected lifestyle choices) which helped residents to feel secure in their surroundings and improve adherence to medical treatment,^19^ while preventing further worsening harmful behaviours, such as the continuing use of drugs and alcohol.^22^ Previous research emphasises that understanding and addressing the complexities of the individual is essential within palliative and end of life care to ensure the delivery of personalised, holistic services.^11 24^

Two interventions and models of care were cost saving.^16 23^ However, others were time consuming to undertake, requiring both investment and dedication from professionals to integrate their newly learnt skills into practice. New interventions sometimes came at the expense and compromise of professionals fulfilling their usual routine tasks.^18^ The role of a healthcare navigator^20^ was recognised as causing a large workload for one person to manage; additionally, its lack of funding highlighted another potential barrier to sustaining the role and maximising its impact long-term.

## Discussion

We identified several pivotal interventions and models of care which were successful for optimising the delivery of palliative and end of life care to homeless adults, and improving their outcomes. These key components are: advocacy; multidisciplinary communication and collaboration; professional education; and community-based, rather than institution-based (hospital or inpatient hospice), care.

Prior evidence supports our findings. A systematic review by Ahmed *et al*^25^ identified that a lack of palliative care knowledge and education among health and social care staff is a severe limitation to providing support to the homeless when living with a terminal illness. Our review shows that educational programmes are beneficial for improving palliative care delivery to homeless adults in the community. The HEARTH study conducted by Crane *et al*^26^ evaluated the success of specialised primary care services to deliver healthcare to the homeless; it emphasised that homeless adults felt most trusting of healthcare providers working within specialist homeless services, tailored to meet their complex needs. Cook *et al*^27^ identified that homeless adults often have significant comorbidities while living with concurrent addiction, meaning their palliative and end of life care needs often differ from the general population. Our review adds to the findings of both Cook *et al* and the HEARTH study and recognises that flexible, holistic, multidisciplinary palliative care is paramount in addressing the trimorbid elements (poor physical health, poor mental health and substance use conditions) influencing palliative care needs among the homeless community.

Somerville^4^ emphasises that homelessness comprises physiological deprivation (lack of bodily comfort or warmth); a pivotal factor needing to be addressed. The research we identified demonstrated gaps in addressing this; for example, some homeless adults were refused access into homeless shelters due to age, leaving them destitute and in distress, despite living with a terminal illness. Two hostels had an upper age limit of 65 years old,^18^ restricting the support accessible to them, yet palliative care provision is most often associated with older patients. 27.2% of adults over 65 identify as homeless,^28^ yet limiting access to hostels where palliative needs can be addressed, due to chronological age, is a significant structural hurdle.

Somerville also states that homelessness involves ontological deprivation relating to a lack of rootedness in the world.^4^ This lack of rootedness was particularly evident for some groups; for example, 25% of transgender adults experience homelessness in Britain,^29^ yet only one study included transgender participants within their study population.^23^ Homeless services often fail to support transgender adults culturally; these individuals are less likely to be accepted into shelter-based accommodation, and due to fear of discrimination and lack of understanding, transgender adults have increasing mistrust in health and social care providers.^30^ The Office for National Statistics^31^ reinforce our findings and document that other homeless populations are also under-recognised, including women and ethnic minorities. Black ethnicities are three times, and mixed race are two times, more likely to be experiencing homelessness than white adults.^28 32^ However, inclusion of these ethnicities which are most prevalently affected by social inequalities are limited in research. Hidden homelessness may explain why these subgroups are limited in inclusion within this review. Hospices and hostels must be inclusive within their policies; it is essential that they adopt safe, stable, and welcoming environments to deliver palliative and end of life interventions and models of care to all demographics of homeless adults.

There are more gaps identified in our review. We found that healthcare needs were not always addressed; in one study, only 57.1% of terminally ill homeless patients admitted into a hospice received a palliative consult during their admission.^23^ Optimising medical comorbidities, through liaison with medical staff and external agencies (mainly primary care providers), was a significant challenge faced by hostel staff to provide total palliative symptom control.^18^

Furthermore, the ability to read and write was a crucial requirement of the homeless adults involved within two interventions,^16 24^ yet one-third of homeless adults have no educational qualifications.^28^ Both literacy and language barriers can make it difficult to engage with homeless adults directly within health and society. Application of structural interventions within health and social care policy, including access to advocates and translators, is essential to overcome these barriers.

We also recognised that the sustainability of some interventions was uncertain. Frequent staff turnover within hostels can affect the long-term impact of newly implemented educational programmes^19^; this risks losing knowledgeable staff and the initial successes achieved in improving palliative care delivery to hostel residents.^19^ We recommend that hostel and hospice staff participation in these educational programmes is made compulsory within their job specification. This will foster permanency of these interventions and models of care within practice; we found one instance of this which was very successful.^18^

This systematic review has strengths and limitations. We have advanced understanding on the interventions and models of care used for homeless adults needing palliative and end of life care, while also considering how well they work in achieving this. The nine included studies have been assessed to be of intermediate methodological quality overall and credible in their findings. However, the synthesis of this review demonstrates that there is a real paucity of research specifically relating to the availability of interventions and models of care used in the delivery of palliative and end of life care to homeless adults. A lack of quantitative data meant that we were unable to numerically quantify the effectiveness of identified palliative and end of life interventions and models of care. We synthesised data, mainly qualitative, from the perspectives of professionals involved in the provision of the palliative and end of life care to homeless adults. Rarely was the viewpoint directly obtained from homeless adults regarding their individual experiences of these interventions and models of care; this is a pertinent barrier which must be addressed in future research.

This paper followed guidance from the CRD^12^ for conducting a systematic review. Studies were limited to those published in the English language and the search strategy did not include bibliographic searching or grey literature; this may have prevented identification of unpublished documentation on interventions and models of care used within current health and social care practice. Only one author had undertaken the quality assessment of individual studies; this may have introduced bias into our methodology. Many studies were conducted in the USA, yet homelessness is a global finding; this limits the generalisability of our results within other cultural contexts. Most studies had small sample sizes which highlights the difficulty in recruiting homeless adults for participation in research.

### Implications

Recent statistics document the rapid rise in the number of people experiencing homelessness^2^: it is imperative that we identify and implement effective palliative and end of life interventions and models of care into practice for homeless adults immediately. This will reduce health inequalities and promote equitable and accessible care to all, regardless of housing status.

All studies in this review addressed at least one of the critical components of homelessness according to the philosophy of Somerville.^4^ However, most failed to equally address and optimise them in their totality. The in-reach support model^18^ was the most valuable approach out of all the studies assessed, encompassing a holistic and inclusive approach to support the palliative and end of life needs of homeless adults within the hostel environment. It placed great emphasis on the optimisation of the medical, psychological, social and spiritual aspects of care for homeless adults, including addiction. Its methodological rigour was of high quality and, matched with the success of the model, we recommended that employment in wider health and social care practice is fulfilled to support the homeless population needing palliative and end of life care.

Gaps in the delivery of palliative and end of life care to homeless adults have been highlighted and we indicate where further direction is needed. We acknowledge that there is no specific definition of homelessness; however, perspectives from adults experiencing other types of homelessness, besides shelter-based living, must be of greater focus in future research. The impact of palliative and end of life interventions and models of care must also be sought from homeless women, older adults, LGBTQ+ and ethnic minorities who are underrepresented in the research.

## Conclusion

There are key components that help optimise support for homeless adults needing palliative and end of life care: advocacy; multidisciplinary working; professional education; and care in the community. Most studies focused on the professionals involved in the care of homeless individuals; few studies included the voices of those experiencing homelessness. Future research must include the perspectives of those who are homeless, build on the components which we know work, and address sustainability of these interventions and models of care.

## supplementary material

10.1136/spcare-2024-004883 online supplemental table 1

10.1136/spcare-2024-004883 online supplemental table 2

## Data Availability

All data relevant to the study are included in the article or uploaded as supplementary information.
