# Supplementary material for: Destitute and dying: interventions and models of palliative and end of life care for homeless adults – a systematic review
Source: BMJ Support Palliat Care. 2024 Aug 17;14(e3):e004883. doi: 10.1136/spcare-2024-004883 (PMC11672054; doi:10.1136/spcare-2024-004883)
Supplement: online supplemental table 1 [file spcare-14-e3-s001.pdf]

Supplementary Table 1: Quality assessment of included studies using the Quality Assessment Tool for Qualitative Studies.

	Abstract and title	Introduction and aims:	Method and data:	Sampling:	Data analysis:	Ethics and bias:	Results:	Transferability or generalizability:	Implications and usefulness:	Quality Approved Total score allocation Maximum score = 36
**Experiences of Homeless Recipients of Social Model Hospice Care: A Photovoice Exploration** Jensen et al.	4	4	3	3	4	3	3	3	3	30/36
**Effect of an End-of-Life Planning Intervention on the Completion of Advance Directives in Homeless Persons** Song et al.	4	4	4	3	4	3	3	2	3	30/36
**Effect of a medical student-led end-of-life planning intervention in completion of advanced directives among homeless persons** Coulter, A. M. Z.	4	3	3	3	1	2	3	3	2	24/36
**Shelter-based palliative care for the homeless terminally ill** Podymow, Turnbull and Coyle.	4	2	3	4	3	1	4	4	4	29/36
**Supporting homeless people with collaborative palliative and end-of-life care** Speight and Lyons	1	2	2	1	2	1	3	3	2	17/36
**Assessing the impact of a health navigator on improving access to care and addressing the social needs of palliative care patients experiencing homelessness: A service evaluation** Robinson et al.	4	3	3	1	3	4	3	2	3	26/36
**The benefits and challenges of embedding specialist palliative care teams within homeless hostels to enhance support and learning: Perspectives from palliative care teams and hostel staff.** Armstrong et al.	4	4	4	3	4	4	4	3	3	33/36
**Evaluation of training on palliative care for staff working within a homeless hostel** Shulman et al.	4	4	4	3	4	3	4	3	3	30/36
**Palliative Education and Care for the Homeless (PEACH): A Model of Outreach Palliative Care for Structurally Vulnerable Populations.** Buchanan et al.	2	2	2	2	2	1	3	3	3	20/36

Key. Good =4, Fair =3, Poor =2, Very poor=1
