# Supplementary material for: Destitute and dying: interventions and models of palliative and end of life care for homeless adults – a systematic review
Source: BMJ Support Palliat Care. 2024 Aug 17;14(e3):e004883. doi: 10.1136/spcare-2024-004883 (PMC11672054; doi:10.1136/spcare-2024-004883)
Supplement: online supplemental table 2 [file spcare-14-e3-s002.pdf]

Supplementary Table 2: outcomes and evidence of effectiveness of included interventions and models of care.

Reference, duration, location. study type	Outcomes	Evidence of how well it worked	Evidence of how well it worked
**Effect of an End-of-Life Planning Intervention on the Completion of Advance Directives in Homeless Persons**<br>Song et al.<br><br>Conducted: November 2007-August 2008.<br><br>Location: USA.<br><br>Study type: randomised control trial.	• Primary outcome- completion of an advance directive within 3 months of enrolment into the study.<br>• Advance directives were assessed for their legibility and legality status by multiple independent investigators who were blinded to intervention groups.<br>• 70/262 completed the advance directive. 2 were not counted due to illegibility.<br>• Higher completion rate in the counsellor guided intervention group- 37.9% compared to the self-directed group-12.8%. p value <0.001.	++	• Aimed to maintain autonomy and dignity of the vulnerable homeless adults upon documentation of their preferences for end-of-life care.<br>• Help was given to the self-guided treatment arm in the form of assistance for reading and writing, and provision of pens and reading glasses.<br>• More than one quarter successfully completed an advance directive when provided with the opportunity, matched similarly to completion in the general population with rates of 15-30%.<br>• Acknowledged that literacy barriers are an issue to completion, especially in the self-directed group. This was a hurdle easier to address within the counsellor assisted intervention.<br>• Interactive and personalised intervention.
**The benefits and challenges of embedding specialist palliative care teams within homeless hostels to enhance support and learning: Perspectives from palliative care teams and hostel staff**<br>Armstrong et al.<br><br>Conducted: December 2018- June 2020.<br><br>Location: UK.<br><br>Study type: exploratory qualitative study.	• Hostel residents reported positively feeling cared for by staff.<br>• Staff felt empowered following introduction of homeless champions; there was a shift in mindset upon the development of holistic palliative ethos within their practice.<br>• Homeless champions improved interagency and multiagency communication and collaboration.<br>• Mental health addressed was through introduction of death café and vigil.<br>• Residents were less inclined to blame staff for deaths of their peers; staff and residents supported each other through the grieving process.	+++	• High staff turnover could be a barrier to sustainability.<br>• Beneficial for staff and residents, increased rapport, and trust.<br>• Person centred approach to care adopted.<br>• Some hostels were wet hostels allowing alcohol use on site which enhanced inclusivity.<br>• Death café and vigil project initiated in the project to encourage openness around death and dying and honour the bereaved. This destigmatised death and the dying process.<br>• Bereavement support given to staff and residents by homeless champions- useful, thoughtful and person centred.<br>• Timely service comes at a trade-off of other parts of the staff workload.<br>• Managerial support from the hospice is crucial to allow time away from the hospice to work as champions at the hostel.<br>• Inclusion health in reach services need to be embedded in all hostels to optimise care and reduce workload of palliative care champions.
**Assessing the impact of a health navigator on improving access to care and addressing the social needs of palliative care patients experiencing homelessness: A service evaluation**<br>Robinson et al.<br><br>Conducted: July 2020-July 2021.<br><br>Location: Canada.	• One social worker assisted a maximum of 50 homeless adults within their service at any one time.<br>• 2007 activities completed by the healthcare navigator for adults experiencing homelessness and needing palliative care support.<br>• A focused approach to the social determinants of health was undertaken to facilitate equitable care at the end of life.	++	• Valuable for improving care for the vulnerable.<br>• Collaboration optimises care. Better integration of social services with healthcare services, reducing fragmentation.<br>• Timely and labour-intensive intervention to undertake but was worthwhile.<br>• High workload for one person to achieve.<br>• Structural barriers- funding to sustain the role of the healthcare navigator, and employment to multiple social workers as healthcare navigators needed to maximise its potential impact.

Study type: Service evaluation.			
**Supporting homeless people with collaborative palliative and end-of-life care**<br>Speight and Lyons.<br><br>Conducted: 2021.<br><br>Location: UK.<br><br>Study type: Service improvement.	• Front line staff were engaged to deliver advance care planning with homeless adults; they were understanding of the importance of advance care planning and increased its uptake and completion.<br>• Increased number of homeless individuals dying with dignity in preferred place of care with the right support.<br>• 3 patients died in preferred place of care; 4 patients were supported in accommodation.<br>• Improved front line staff knowledge on the identification of deteriorating patients, palliative and end of life care.	+	• Interventions delivered online and face to face to improve accessibility.<br>• Upskilling of staff was empowering and boosted confidence.<br>• Increased referral of patients identified as having deteriorating health needs by front line staff.<br>• Earlier identification of deteriorating health meant quicker access to clinical support. This helped in the stabilisation and treatment of ill health and reduced further preventable deterioration in health.<br>• Increased signposting and engagement between staff and patients.<br>• Reduction in staff fear on talking directly with homeless adults about end of life and knowing what actions to take.
**Shelter-based palliative care for the homeless terminally ill**<br>Podymow, Turnbull and Coyle.<br><br>Conducted: June 2001-August 2003.<br><br>Location: Canada.<br><br>Study type: retrospective cohort study.	• Hostel staff were responsible for supervised provision of medication which improved compliance in the homeless population.<br>• No increase in substance abuse.<br>• Continuity of care in the terminal phase was achieved.<br>• Homeless adults were reunited with family when wanted, and where possible.<br>• 57% had palliative care consult.<br>• 82% died in hospice; 18% of homeless adults transferred to the emergency department for further symptom control at their request.<br>• End of life issues were discussed, and religious needs met.	+	• Cost saving: hospice-based care cost less than predicted costs of healthcare from traditional care provision, based on the judgements from an expert panel.<br>• Inclusive- transgender residents enrolled and residents with addiction.<br>• No QOL assessment but gratitude was expressed by patients.<br>• Harm reduction approach helped to reduce substance misuse and improve homeless adult compliance in taking prescribed medications (>80%).
**Effect of a medical student-led end-of-life planning intervention in completion of advanced directives among homeless persons**<br>Coulter.<br><br>Conducted: 2016.<br><br>Location: USA.<br><br>Study type: qualitative study.	• 9/10 homeless adults who attended focus groups signed up to complete an advance directive.<br>• 88.8% completion rate.	++	• Encouraged patient agency and autonomy.<br>• Free completion of the living will.<br>• Literacy barriers arose due to the need for reading the information worksheet on advanced directives.<br>• No females enrolled in the intervention, yet they are a large part of the homeless population.<br>• 1 medical student volunteer- more would be needed to ensure the time and investment does not impact studies.
**Evaluation of training on palliative care for staff working within a homeless hostel**<br>Shulman et al.<br><br>Conducted: 2018.<br><br>Location: UK.	• All sections of the course were interesting and useful according to hostel staff.<br>• Knowledge of palliative care and services available was most improved amongst hostel staff.<br>• Staff gained confidence in supporting their clients; also gained knowledge on how to access support, and how to give support to residents.	+	• Hostels focus on recovery, but this training has also emphasised the potential for a radical shift to palliation for some residents.<br>• High staff turnover at the hospice- palliative training seemed successful but if the staff move on to other hostels, new staff will lack the benefits of the training and potential palliative patients will be denied additional support from those trained.<br>• 2-day course felt to be too long by some staff.

Study type: mixed methods study with pre and post training data collection.	• Hostel staff more open to confront ill health and talk about it with the clients.<br>• Enhanced self-awareness of staff to support their own health and wellbeing. Work related stress was also slightly improved.<br>• Improved attitudes and openness of hostel staff to supporting dying patients and accepting of these dying patients remaining in the hostel. However, some staff did not want the hostel to turn into a hospice environment.		• Training tools provided lacked content on exploring the insights and wishes of patients. No advance care planning awareness was included.<br>• System level change is hard to achieve structurally in the hostel environment to enable palliative provisions.<br>• Multiagency working encouraged. But lack of information sharing about the resident's information to different agencies was noted, and issues arose amongst hostel staff who were unsure how to access support from other outside organisations and arrange support for their residents.<br>• Staff reported a lack of clarity of their job role due to additional training and expectations from course.
**Experiences of Homeless Recipients of Social Model Hospice Care: A Photovoice Exploration**<br>Jensen et al.<br><br>Conducted: January-August 2020.<br><br>Location: USA.<br><br>Study type: qualitative case report.	• Patient reported outcomes assessed including: physical location to receive care; community involvement in care; spiritual needs addressed via access to a chaplain.<br>• Allowed to bring pets on site.<br>• Family re-connected with residents.<br>• Residents felt "at home" and cared for by staff and other residents.<br>• Harm reduction strategy allowed residents to die with dignity in a comfortable, peaceful location.<br>• Healthcare needs and medication was provided by certified nurse's assistants.<br>• Certified nurse' assistants were onsite 24/7 and befriended residents; this created a sense of community and connection which made residents feel more confident in their caregivers.	++	• Residents had a physical location to receive care- grateful for their own space and private restroom.<br>• Financial assistance from local community and acts of goodwill such as food deliveries from local hospitality.<br>• Person centred care made possible by the philanthropy of the community and social model of this initiative.<br>• Sense of community and belonging felt by the residents towards each other- all supporting and caring for each other.<br>• Effective end of life care for dying residents, innovative model and education provided to the local community of the concept of social model hospice upon engagement and recruitment of volunteers and participation in local community outreach events to raise awareness.<br>• Overcomes housing and care giver insecurity for people who need hospice services.
**Palliative Education and Care for the Homeless (PEACH): A Model of Outreach Palliative Care for Structurally Vulnerable Populations**<br>Buchanan et al.<br><br>Conducted: 2014- to present.<br><br>Location: Canada.<br><br>Study type: service evaluation.	• Social service workers and community workers could directly refer to the PEACH model and at the earliest opportunity.<br>• Education was a priority- active role in teaching and offering electives to medical students.<br>• Reflective practice was encouraged for all professionals involved which helped them to explore ways in reducing barriers faced by clients in their practice and beyond.<br>• Advocacy was a key component of the PEACH programme- high quality, early and integrated palliative care for vulnerable patients. Advocating for food, housing, healthcare.<br>• Advocating at the population level to call for societal change was undertaken via teaching and engagement with local government to tackle the issues arising around homelessness and poverty.<br>• Focused on research on the palliative needs of homeless individuals.	++	• Innovative model which was highly tailored in providing community palliative care to meet the unique and complex health and social needs of homeless adults.<br>• Dignity and compassionate care reduced suffering- ethos of a harm reduction approach,<br>• Model that provided equitable access to palliative care.<br>• Harm reduction approach was beneficial for homeless adults with addiction.<br>• Grief circles and bereavement support improved wellbeing for professionals involved in the model.

	• Support was provided on funeral arrangements, leisure activities and reconnecting homeless adults with family members.<br>• Grief circles encouraged all professionals involved in the programme to come together and support one another in grieving a client's death-		

Key: - did not work, + worked slightly, ++ worked moderately, +++ worked well
